# Metabolic syndrome, adiposity, diet, and emotional eating are associated with oxidative stress in adolescents

**DOI:** 10.3389/fnut.2023.1216445

**Published:** 2023-09-12

**Authors:** Sonia L. Ramírez-Garza, Emily P. Laveriano-Santos, Juan J. Moreno, Patricia Bodega, Amaya de Cos-Gandoy, Mercedes de Miguel, Gloria Santos-Beneit, Juan Miguel Fernández-Alvira, Rodrigo Fernández-Jiménez, Jesús Martínez-Gómez, Ana María Ruiz-León, Ramon Estruch, Rosa M. Lamuela-Raventós, Anna Tresserra-Rimbau

**Affiliations:** ^1^Department of Nutrition, Food Science and Gastronomy, XIA, School of Pharmacy and Food Sciences, Institute for Nutrition and Food Safety Research, University of Barcelona, Barcelona, Spain; ^2^Consorcio CIBER, M.P. Fisiopatología de la Obesidad y Nutrición, Instituto de Salud Carlos III, Madrid, Spain; ^3^Foundation for Science, Health and Education, Barcelona, Spain; ^4^Centro Nacional de Investigaciones Cardiovasculares, Madrid, Spain; ^5^The Zena and Michael A. Wiener Cardiovascular Institute, Icahn School of Medicine at Mount Sinai, New York, NY, United States; ^6^Hospital Universitario Clínico San Carlos, Madrid, Spain; ^7^Centro de Investigación Biomédica En Red en Enfermedades CardioVasculares, Madrid, Spain; ^8^Department of Internal Medicine Hospital Clinic, Institut d’Investigacions Biomèdiques August Pi i Sunyer, University of Barcelona, Barcelona, Spain

**Keywords:** nutritional status, nutrition assessment, fish, refined cereals, obesity, emotion, anxiety, depression

## Abstract

**Background:**

Metabolic syndrome (MS), a condition related to adiposity and oxidative stress, can develop in adolescence, a critical stage in life that impacts health in adulthood. However, there is scarce scientific research about the relationship between lifestyle factors, emotion management, and oxidative stress in this phase of life.

**Aim:**

To analyze whether nutritional parameters, lifestyle factors, emotion management, and MS in adolescents are associated with oxidative stress measured by the biomarker 8-isoprostane.

**Methods:**

A cross-sectional study was carried out in 132 adolescents (48.5% girls, aged 12 ± 0.48 years) and data were collected on nutritional parameters (anthropometric measurements, biochemical analyzes, and blood pressure), lifestyle factors (physical activity, sleep, and diet), and emotion management (self-esteem, emotional eating, and mood). 8-isoprostane was analyzed in spot urine samples. The study population was categorized in three groups (healthy, at-risk, and with MS) using the International Diabetes Federation definition of MS in adolescents. To capture more complex interactions, a multiple linear regression was used to analyze the association between 8-isoprostane and the aforementioned variables.

**Results:**

Urinary 8-isoprostane levels were significantly higher in the MS group compared to the healthy group (1,280 ± 543 pg./mg vs. 950 ± 416 pg./mg respectively). In addition, univariable analysis revealed positive significant associations between 8-isoprostane and body mass index, waist circumference, waist-to-height ratio, body fat percentage, blood lipid profile and glucose, emotional eating, and refined cereal intake. Conversely, a negative significant association was found between 8-isoprostane and sleep duration and fish intake. The multiple linear regression analysis revealed associations between 8-isoprostane and LDL-c (*β* = 0.173 value of *p* = 0.049), emotional eating (low *β* = 0.443, value of *p* = 0.036; high *β* = 0.152, value of *p* = 0.470), refined cereal intake (*β* =0.191, value of *p* = 0.024), and fish intake (*β* = −0.187, value of *p* = 0.050).

**Conclusion:**

The MS group, LDL-c, emotional eating, and high refined cereals and low fish intakes were associated with higher levels of oxidative stress in an adolescent population.

## 1. Introduction

Adolescence is a critical stage in life, when healthy or risk behaviors of adulthood are first established, and social and emotional development affects decision-making and behavior (1, 2). The development of obesity or cardiovascular diseases can be accompanied by the emergence of mental health disorders such as depression, eating disorders, and low self-esteem (3, 4). Furthermore, oxidative stress has been linked with depression (5, 6), whereas a healthy dietary pattern is associated with good mental health (1, 7).

Obesity and metabolic syndrome (MS) are multifactorial diseases estimated to affect 5% of the global adolescent population in 2020 (8). MS is a cluster of risk factors for cardiovascular disease, type 2 diabetes, or prediabetes. These conditions include high blood pressure (BP), high triglycerides, and abdominal obesity, the latter being the key factor for MS diagnosis in adolescents (9). When these pathological triggers are present chronically, a dysfunctional mitochondria could be developed, which can cause an oxidative stress (10, 11), increasing its levels of biomarkers such as 8-isoprostane (12, 13). Isoprostanes are biosynthesized by a free radical-catalyzed peroxidation primarily from arachidonic acid, but also from docosahexaenoic and eicosapentaenoic acids (14, 15) and 8-isoprostane serves as a biomarker of oxidative stress status (16, 17). Due to the scant research on the relationship between 8-isoprostane and MS in adolescents, it is important to study this research topic and its association with factors such as lifestyle, emotion, and nutrition.

The aim of the present study was to analyze the association of nutritional parameters, lifestyle, emotion management, and MS with oxidative stress, measured by 8-isoprostane levels, in a cohort of adolescents enrolled in the SI! Program for Secondary Schools in Spain.

## 2. Materials and methods

The present cross-sectional analysis was performed in a sub-sample of participants enrolled at baseline (1st grade of Secondary School) in the SI! Program for Secondary Schools in Spain (clinical trials register: NCT03504059); all details have been previously published (18). A schematic view of the design and different phases of the present study is provided in Supplementary Figure S1.

### 2.1. Anthropometric measurements

Measurements were obtained after overnight fasting by trained nutritionists. Height was measured with a Seca 213 portable stadiometer (0.1 cm of precision). Body weight, body fat percentage, and skeletal muscle percentage were measured by bioelectrical impedance analysis (OMRON BF511 with 0.1 Kg precision), with participants wearing light clothes and no shoes. Body mass index (BMI) was calculated as body weight divided by height squared (kg/m^2^). Waist circumference (WC) was measured three times with a Holtain tape to the nearest 0.1 cm. Waist-to-height ratio (WHtR) was calculated as WC divided by height. BMI, WC, and WHtR were adjusted for age and sex to obtain z-score values (19, 20).

### 2.2. Biochemical analyzes

Biochemical blood analysis was performed by trained nurses using samples taken early in the morning after overnight fasting. Glucose, triglycerides, total cholesterol, high density lipoprotein cholesterol (HDL-c), and low density lipoprotein cholesterol (LDL-c) were determined in capillary blood samples using the Cardio Check Plus device and PTS Panels test strips (21). Fasting spot urine samples were collected in the morning. The urine was aliquoted and stored at −80°C for subsequent analysis. 8-Isoprostane concentration in urine was determined using the ELISA Kit protocol (Cayman Chemical, Ann Arbor, MI, United States, Item No. 516351). Creatinine was measured by the validated Jaffé alkaline picrate method (22). 8-Isoprostane levels were normalized by creatinine and the results were expressed as pg./mg creatinine.

### 2.3. Blood pressure

BP was measured with an OMRON M6 monitor with 2–3 min intervals between measurements. When the differences between the measurements were less than 10 mmHg for systolic blood pressure (SBP) and less than 5 mmHg for diastolic blood pressure (DBP), two measurements were taken; otherwise, a third reading was performed. Average values were calculated for the final SBP and DBP, which were adjusted for age, sex, and height to obtain percentile and z-score values (23, 24).

### 2.4. Physical activity and sleep characteristics

Moderate and vigorous physical activity levels and sleep duration were estimated from data from the triaxial accelerometer (Actigraph wGT3X-BT) worn on the non-dominant wrist for seven consecutive days. Activity information was considered valid if data were available for a minimum of 4 days, with at least 600 min of wear time per day. Physical activity intensities were estimated using the cut-off points of Chandler et al. (25). The sleep algorithm proposed by Sadeh et al. (26) was used to obtain sleep duration (total of hours asleep), sleep efficiency (number of sleep minutes divided by the total number of minutes the subject was in bed), awakenings (the number of awakenings per night), and time spent awake after initially falling asleep (the average length of all awakening episodes in minutes). All measurements were obtained using ActiLife software (Version 6.13.4, LLC).

### 2.5. Emotion management

To assess emotion management, three different subscales were used, namely “self-esteem,” “emotional eating” (EE), and “mood,” which were measured through validated questionnaires filled out by the participants. Four response categories on the Likert scale were used for self-esteem and EE items, and five response categories for mood items.

Self-esteem was assessed with five items of the Child Health and Illness Profile–Adolescent Edition test (CHIP-AE) (27). An example of an item is, “I like the way I am,” to which there are four response options: strongly disagree = 1, disagree = 2, agree = 3, and strongly agree = 4. Thus, higher scores designate better health-related outcomes. The score was obtained by calculating the mean scores. Internal reliability (Cronbach’s ⍺) was 0.797.

EE was assessed with three items of the Three-Factor Eating Questionnaire-R18 (Tfeq-Sp) (28). An example of an item is, “When I feel anxious, I find myself eating,” to which there are four response options: definitely false = 1, mostly false = 2, mostly true = 3, and definitely true = 4. Higher scores indicate greater levels of EE. Scores were categorized as “No EE,” “Low EE,” and “High EE.” The “No EE” category reflected a score of 3. To create the “Low EE” and “High EE” categories, a median split was used (excluding the “No” category), and scores below the median were categorized as “Low EE” and scores above the median as “High EE” (4). Internal reliability (Cronbach’s ⍺) was 0.791.

Mood was assessed with a six items of the validated FRESC (Factors de Risc en Estudiants de SeCundària) lifestyle risk-factor survey for secondary school students (29). An example of an item is, “I am too tired to do anything,” with the following response options: never, almost never, sometimes, frequently, and always. The variable was dichotomized, whereby the response “always” or “frequently” to three or more of the six items indicated a negative mood state, whereas a positive mood state was assigned for the other responses. Internal reliability (Cronbach’s ⍺) was 0.673 as reported by Ahonen et al. (30).

### 2.6. Dietary data

A validated semi-quantitative food frequency questionnaire with 157-items was filled out by the families of the participants to provide information about their dietary habits from the previous year (31, 32). The items were organized by food groups and the response categories were as follows: never or almost never, 1–3 per month, 1 per week, 2–4 per week, 5–6 per week, 1 per day, 2–3 per day, 4–6 per day, and 6 or more per day. This questionnaire results were analyzed using Evaldara software and the food composition tables of the Centro de Enseñanza Superior de Nutrición y Dietética and adjusted for total energy intake (33, 34).

### 2.7. Metabolic groups

The study population was categorized in three metabolic groups (healthy, at-risk, and MS) as defined by the International Diabetes Federation (9). Those in the MS group had abdominal obesity (≥ 90th percentile of WC) plus two or more clinical symptoms: ≥ 150 mg/dL of triglycerides or < 40 mg/dL of HDL-c or ≥ 100 mg/dL of blood glucose or high BP (SBP ≥ 130 mm Hg or DBP ≥ 85 mm Hg). The at-risk population included participants with MS symptoms but not enough to belong to the MS group. Those in the healthy population showed none of the aforementioned symptoms. After the participants were categorized, a randomized sampling from each group was carried out to define the at-risk and healthy groups. Supplementary Figure S2 shows a schematic view of the process followed in the different phases of defining the study groups.

### 2.8. Statistical analyzes

A minimum of 118 participants were required to provide 95% power of test and a significance level of 5% when performing multiple regression. Given that 44 participants presented MS, a total of 132 participants were enrolled to achieve a ratio of 1:1:1 for the three groups (44 in each); more details are provided in Supplementary Figure S2.

A seven-step statistical process was followed; a value of *p* ≤0.05 was considered statistically significant. First, a 98% winsorizing technique was used to minimize the influence of outliers. Second, numerical variables without prior standardization (i.e., adjusted for age, sex, and height) were standardized (z-scores) prior to the statistical analysis. Third, the normality of variables was determined by the Kolmogorov–Smirnov test. Fourth, to estimate differences, a chi-square test was used for categorical variables, an analysis of variance (ANOVA) for parametric numerical variables, and a Kruskal–Wallis test for non-parametric numerical variables, whereas Dunn–Bonferroni correction was used to ascertain differences between the metabolic groups. Fifth, a simple linear regression was used to identify the association between 8-isoprostane levels and each studied variable. Sixth, principal component analysis was used to eliminate collinearity among variables. Finally, to capture more complex interactions between different factors, a multiple linear regression model was generated. All statistical analyzes were conducted using R software version 4.1.0 (R Studio, 250 Northern Ave, Boston, MA, United States).

## 3. Results

The present study enrolled 132 adolescents (48.5% girls) aged 12 ± 0.48 years. The healthy group had the highest percentage of girls (56.8%), and the MS group the highest percentage of boys (63.6%) (Table 1).

**Table 1:** Description and comparison of the metabolic groups.

	Healthy	At-risk	Metabolic syndrome	Value of *p* †
*n*	44	44	44	
Boys (%)	43.2	50.0	63.6	0.131
Anthropometric variables				
BMI z-score	0.26 ± 0.51 *	1.29 ± 0.87 *	2.42 ± 0.49 *	<0.001
WC z-score	−0.13 ± 0.57 *	0.86 ± 0.75 *	1.77 ± 0.28 *	<0.001
WHtR z-score	−0.48 ± 0.58 *	0.47 ± 0.9 *	1.63 ± 0.35 *	<0.001
Body fat (%)	19.9 ± 5.46 *	27.3 ± 9.0 *	36.7 ± 4.40 *	<0.001
Skeletal muscle (%)	35.4 ± 2.41 *	33.6 ± 3.53 *	30.7 ± 2.23 *	<0.001
Biochemistry analysis:				
Total cholesterol (mg/dL)	147 ± 24.1	148 ± 39.9	164 ± 42.7	0.050
Blood glucose (mg/dL)	91 ± 6.8 *	104 ± 11.9 *	112 ± 9.5 *	<0.001
Triglycerides (mg/dL)	66 ± 11.2 ^B^	91 ± 37.7^C^	154 ± 96.6 ^B, C^	<0.001
HDL-c (mg/dL)	66 ± 14.2 ^A, B^	54 ± 17.4 ^A^	50 ± 15.0 ^B^	<0.001
LDL-c (mg/dL)	68 ± 20.6	76 ± 34.4	83 ± 36.9	0.079
Urinary 8-isoprostane (pg/mg)	950 ± 416 ^B^	1,133 ± 447	1,280 ± 543 ^B^	0.005
Blood pressure:				
SBP z-score	−0.03 ± 0.84 ^A, B^	1.18 ± 0.89 ^A^	1.22 ± 0.93 ^B^	<0.001
DBP z-score	0.24 ± 0.80 ^A, B^	0.90 ± 0.96 ^A^	1.15 ± 1.02 ^B^	<0.001
Physical activity and sleep:				
MVPA (minutes)	72.6 ± 23.4	69.4 ± 27.8	71.4 ± 18.6	0.813
Sleep duration (hours)	7.83 ± 0.71 ^B^	7.72 ± 0.74^C^	7.17 ± 0.99 ^B, C^	<0.001
Sleep efficiency (%)	92.9 ± 2.84	92.8 ± 3.02	91.4 ± 3.18	0.059
Awakenings (Frequency)	16.9 ± 5.32	16.0 ± 6.06	14.2 ± 5.15	0.095
Awake length (minutes)	2.03 ± 0.38 ^B^	2.06 ± 0.38^C^	2.63 ± 0.76 ^B, C^	<0.001
Emotion management:				
Satisfaction: self-esteem (1 to 4)	3.6 (1.4–4.0)	3.6 (2.0–4.0) ^C^	3.4(0.8–4.0) ^C^	0.037
Emotional eating				
No emotional eating (%)	59.1	52.3	38.6	0.148
Low emotional eating (%)	18.2	29.5	20.5	0.404
High emotional eating (%)	22.7	18.2	40.9	0.040
Mood				
Positive mood (%)	93.2	90.9	86.4	0.550
Negative mood (%)	6.8	9.1	13.6	
Dietary:				
Energy intake (kcal/d)	2,450 ± 586	2,486 ± 679	2,522 ± 534	0.878
- Carbohydrates (% EI)	39.9 ± 5.98	41.7 ± 6.97	40.5 ± 7.01	0.434
- Total fat (% EI)	40.2 ± 4.88	39.4 ± 6.47	39.8 ± 6.49	0.822
- Protein (% EI)	19.8 ± 3.20	18.7 ± 2.94	19.7 ± 2.90	0.206
Intake by groups of foods:				
Fruits and vegetables				
- Vegetables (s/d)	1.7 (0.0–5.8)	2.0 (0.2–6.1)	2.1 (0.5–6.5)	0.559
- Fruits (s/d)	1.4 (0.1–7.4)	1.8 (0.1–8.7)	1.9 (0.1–6.9)	0.729
Cereals				
- Whole grain (s/d)	0.1 (0.0–1.6)	0.1 (0.0–1.0)	0.1 (0.0–1.3)	0.900
- Refined cereals (s/d)	0.9 (0.0–1.9)	0.8 (0.0–2.6)	0.9 (0.2–1.7)	0.728
Fats and Oil (g/d)	24 (2.9–129)	26.3 (1.4–79)	20.9 (3.1–67)	0.857
Dairy products (s/d)	2.9 (0.7–15.7)	2.4 (0.0–7.0)	2.4 (0.0–6.9)	0.292
Legumes (s/w)	2.5 (0.0–12.0)	2.4 (0.0–28.0)	3.0 (0.0–14.5)	0.131
Eggs (s/w)	3.0 (0.0–7.0) ^A^	3.0 (0.0–6.0) ^A^	3.0 (0.5–7.0)	0.008
Fish (s/w)	3.2 (0.0–6.0) ^B^	2.0 (0.0–6.0) ^C^	1.0 (0.0–6.0) ^B, C^	<0.001
Meat and processed meat (s/w)	13.1 (4.9–31.4)	14.5 (0.9–54.3)	16.9 (4.4–35.0)	0.369
Sugar-sweetened beverages (s/w)	0.7 (0.0–6.0)	0.5 (0.0–6.0)	1.0 (0.0–6.8)	0.913

Numerical variables were standardized prior to the statistical analysis. †value of *p*s from chi-square (%), ANOVA (mean ± SD), and Kruskal-Wallis (median and range) tests. Significant differences (value of p ≤ 0.05) among segments after Bonferroni post-hoc correction; * among all groups; A, between healthy and at-risk groups; B, between healthy and metabolic syndrome groups; C, between at-risk and metabolic syndrome groups. 8-Isoprostane is expressed by pg of 8-isoprostane/mg of creatinine in urine. BMI, body mass index; WC, waist circumference; WHtR, waist-to-height ratio; HDL-c, high-density lipoprotein cholesterol; LDL-c, low-density lipoprotein cholesterol; SBP, systolic blood pressure; DBP, diastolic blood pressure; MVPA, moderate and vigorous physical activity. Intake is expressed in % EI; percentage of energy intake; g/d, grams per day; s/d, serving per day; s/w, serving per week.

The characteristics of the study population and the differences between the three metabolic groups are described in Table 1 and Supplementary Table S1 shows the differences between sexes. Significant differences in all anthropometric measurements and blood glucose levels were observed for all metabolic groups. Triglycerides, HDL-c, 8-isoprostane levels, BP, sleep duration, time spent awake after initially falling asleep, and fish intake differed significantly between the healthy and MS groups. It is worth noting that the adolescents in the healthy group had longer periods of sleep interrupted by shorter awake periods compared to the MS group.

**Figure 1:**
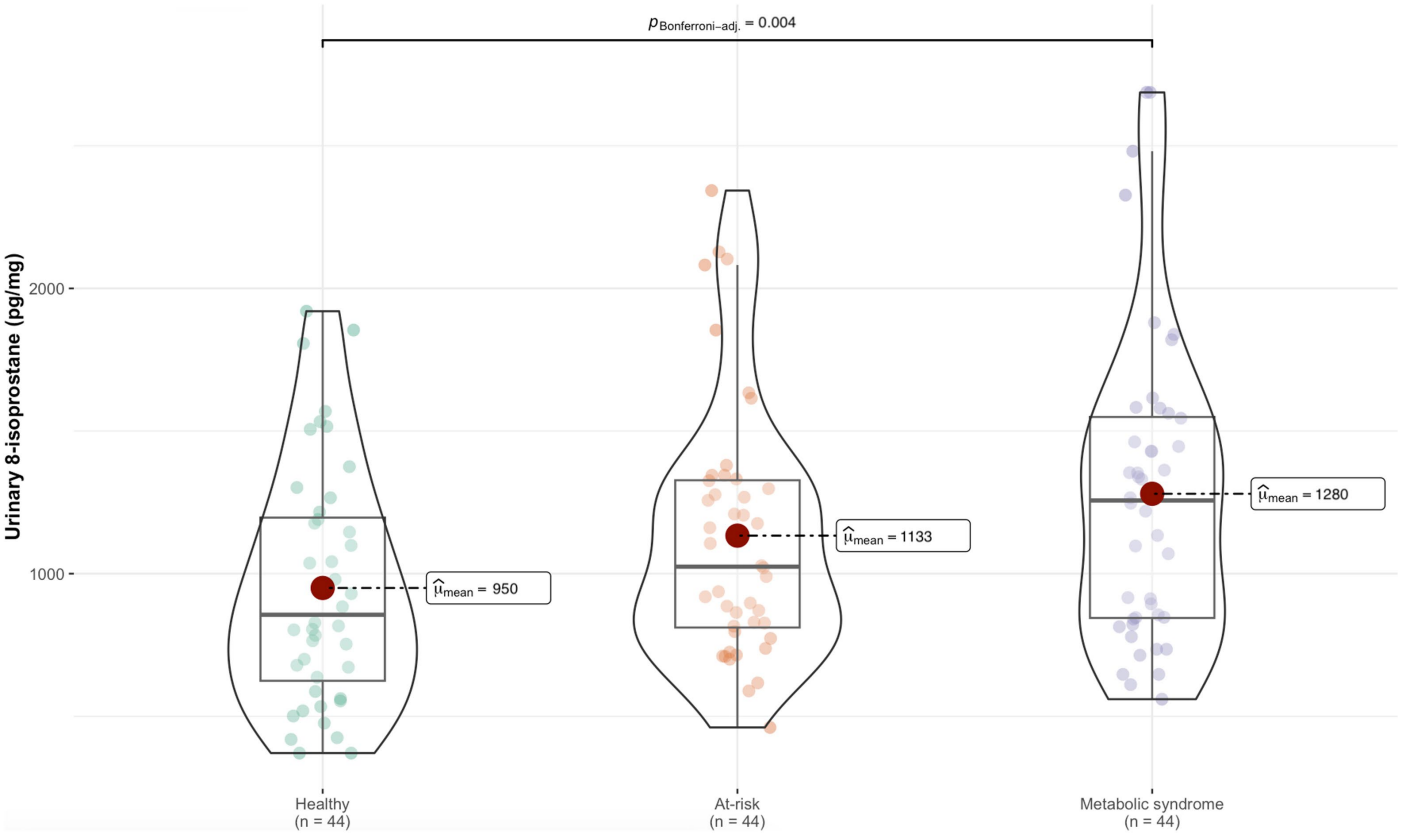
Difference in 8-isoprostane levels between the metabolic groups.

Levels of 8-isoprostane were higher in the MS group than the healthy group (Figure 1). Table 2 shows the association between 8-isoprostane and each nutritional parameter and lifestyle variable. The BMI, WC, and WHtR z-scores, and body fat percentage were positively associated with 8-isoprostane levels, suggesting the biomarker was positively related to adiposity. Similarly, blood glucose, total cholesterol, LDL-c, EE, categorization in the MS group, and refined cereal intake were positively associated with 8-isoprostane; in contrast, sleep duration and fish intake showed a negative association with 8-isoprostane (Table 2). Total cholesterol and z-scores for BMI, WC, and WHtR acted as collinear variables in the dataset under analysis.

**Table 2:** Association of individual variables with 8-isoprostane.

	Coefficient (β)	Value of *p*
*n*		
Boys	0.130	0.293
Anthropometric variables:		
BMI z-score	0.275	<0.001
WC z-score	0.362	<0.001
WHtR z-score	0.348	<0.001
Body fat (%)	0.251	0.004
Skeletal muscle (%)	−0.159	0.068
Biochemistry analysis:		
Blood glucose (mg/dL)	0.217	0.012
Triglycerides (mg/dL)	0.170	0.051
Total cholesterol (mg/dL)	0.234	0.007
HDL-C (mg/dL)	−0.069	0.434
LDL-c (mg/dL)	0.232	0.007
Blood pressure:		
SBP z-score	0.028	0.739
DBP z-score	0.098	0.262
Physical activity and sleep:		
MVPA (minutes)	0.121	0.181
Sleep duration (hours)	−0.236	0.009
Sleep efficiency (%)	−0.087	0.342
Awakenings (frequency)	−0.096	0.292
Awakenings (length in minutes)	0.074	0.414
Emotion management:		
Self-esteem	0.001	0.984
Emotional eating*		
- Low emotional eating (%)	0.599	0.006
- High emotional eating (%)	0.403	0.048
Negative mood*	−0.223	0.448
Metabolic groups*		
- Risk group	0.376	0.070
- Metabolic syndrome group	0.677	0.001
Dietary:		
Energy intake (kcal/d)	0.021	0.806
Carbohydrates (% EI)	0.089	0.295
Total fat (% EI)	−0.024	0.781
Protein (% EI)	−0.048	0.572
Intake by groups of foods:		
Fruits and vegetables		
Vegetables (s/d)	−0.087	0.309
Fruits (s/d)	−0.021	0.810
Cereals		
Whole grain (s/d)	−0.114	0.180
Refined cereals (s/d)	0.208	0.013
Fats and Oil (g/d)	0.095	0.263
Dairy products (s/d)	−0.137	0.108
Legumes (s/w)	−0.079	0.356
Eggs (s/w)	0.012	0.888
Fish (s/w)	−0.318	<0.001
Meat and processed meat (s/w)	0.033	0.696
Sugar-sweetened beverages (s/w)	0.016	0.847

Numerical variables were standardized prior to the statistical analysis. p-values and β are of simple linear regression. BMI, body mass index; WC, waist circumference; WHtR, waist-to-height ratio; HDL-c, high-density lipoprotein cholesterol; LDL-c, low-density lipoprotein cholesterol; SBP, systolic blood pressure; DBP, diastolic blood pressure; MVPA, moderate and vigorous physical activity. Intake is expressed in % EI; percentage of energy intake; g/d, grams per day; s/d, serving per day; s/w, serving per week. *Intercept (β) for the reference categories: no emotional eating (β = −0.246) for emotional eating; positive mood (β = 0.022) for mood; healthy (*β* = −0.351) for metabolic groups.

**Figure 2:**
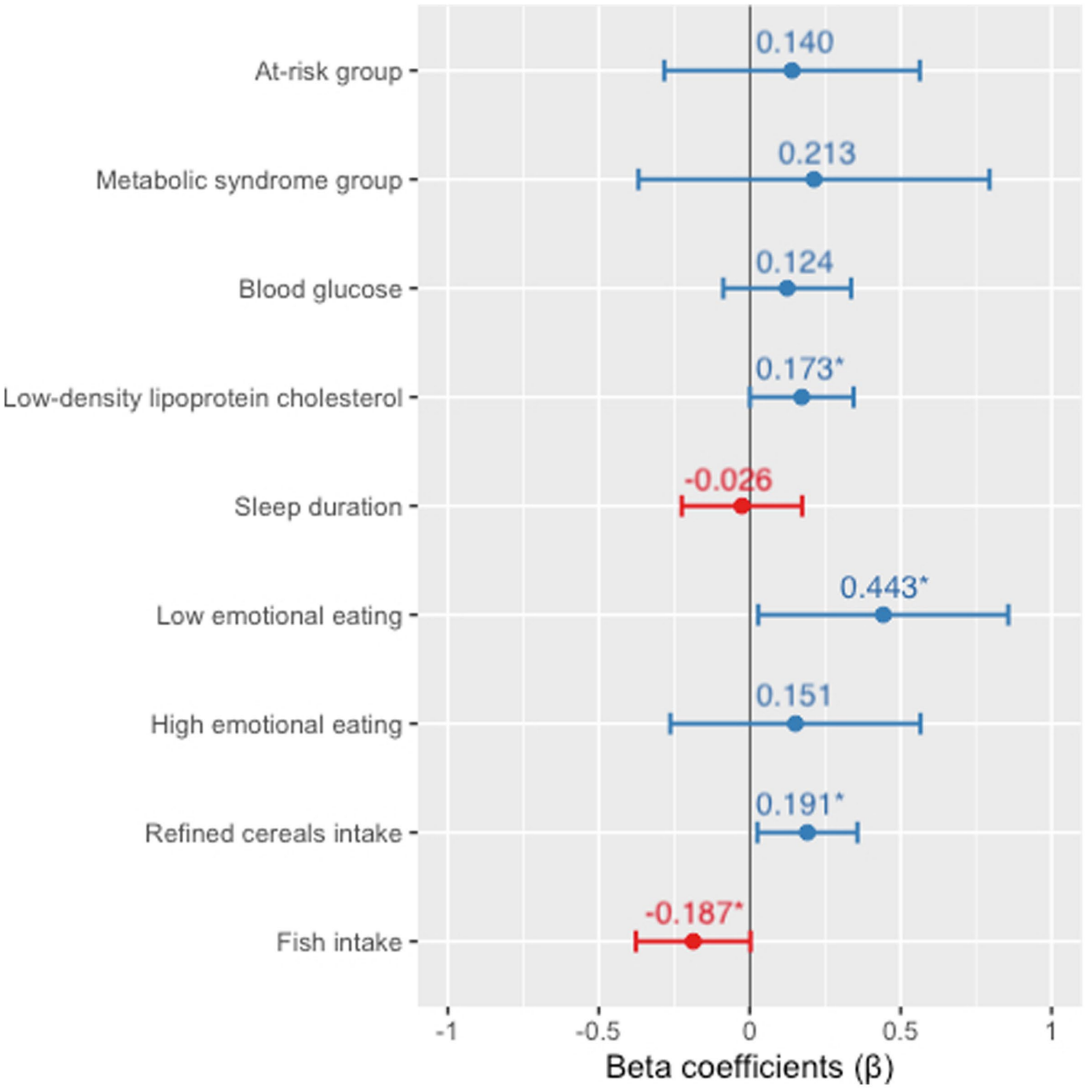
Multiple linear regression model for 8-isoprostane associations. Adjusted for sex and energy intake. Numerical variables are expressed as z-scores. *Statistically significant difference (value of *p* ≤0.05).

Finally, the multiple adjusted linear regression model (Figure 2) showed an association between 8-isoprostane and metabolic group (at-risk *β* = 0.140, value of *p* = 0.513; MS *β* = 0.213, value of *p* = 0.470), blood glucose (*β* = 0.124, value of *p* = 0.247), LDL-c (*β* = 0.173, value of *p* = 0.049), sleep duration (*β* = −0.026, value of *p* = 0.798), EE (low *β* = 0.443, value of *p* = 0.036; high *β* = 0.151, value of *p* = 0.470), refined cereal intake (*β* =0.191, value of *p* = 0.024), and fish intake (*β* = −0.187, value of *p* = 0.050).

## 4. Discussion

Three principal observations were made in the course of the present study. First, 8-isoprostane was associated with an unhealthy metabolic status in the adolescent cohort, being positively related with adiposity (high z-scores for BMI, WHtR, and WC, and high body fat percentage), LDL-c, and MS. Second, an association between EE and 8-isoprostane was found, which to our knowledge has not been previously reported in adolescents. Finally, our results suggest that diet can significantly influence 8-isoprostane levels, which were positively associated with refined cereal intake and negatively associated with fish intake.

Consistent with our results, higher 8-isoprostane levels have been observed in obese children and adolescents compared to those with normal weight (35–37). This biomarker has also been positively correlated with measures of fatness, as well as WC, WHtR, and body fat (38–40). The association we found between oxidative stress, manifested by increased levels of 8-isoprostane, and MS in an adolescent population is also supported by previous studies in children (38, 41, 42) and adolescents (35), which report higher levels of 8-isoprostane in those with symptoms of MS. Moreover, overweight children with MS showed higher 8-isoprostane levels than overweight children without metabolic risk factors (42); it is possible that risk factors such as hyperglycemia, hypertriglyceridemia, presents in MS contribute to the presence of oxidative stress (10). Adolescents with a high BMI were observed to have higher levels of 8-isoprostane if they were insulin-resistant as opposed to insulin-sensitive, but no difference was observed with a low BMI and resistance/sensitivity to insulin (37), suggesting that both, adiposity and risk factors, could conduce to oxidative stress (10, 43). Therefore, it is possible that oxidative stress worsens with obesity, especially when coupled with MS risk factors. In the present study, consistent with the symptoms of MS, LDL-c was positively associated with 8-isoprostane; similar results have been reported in children with excess weight (38), children with diabetes mellitus type 1 (44), and adolescents with insulin resistance (45).

Although a relationship between high EE and MS has been described in adults (4, 46, 47), it remains poorly researched in adolescents. The most closely related study was carried out with adolescents diagnosed with type 1 diabetes, in whom higher EE values were associated with higher levels of HbA1, total cholesterol and LDL-c, which are risk factor of MS (48). On the other hand, an association between EE and obesity, which is the principal characteristic of MS, has been observed in adults (49–55), but has been scarcely studied in adolescents (56).

Since sleeping and physical activity are very important factors in the health of adolescents, we evaluated these variables and consistently with others results, we observed an association between 8-isoprostane and sleep duration (57) and between MS and sleep duration (58), not with physical activity. However, in the multiple linear regression analysis, physical activity or sleep duration were not significant factors in 8-isoprostane level.

As reported in the aforementioned studies, EE is linked with obesity. The association between obesity and 8-isoprostane could therefore explain the relationship found in the present study between EE and 8-isoprostane. The EE questionnaire evaluates the individual’s response to food consumption, focusing on the emotions of anxiety, loneliness, and depression. Previous research has shown a positive association between 8-isoprostane and anxiety as well as depression (59–63), which is in agreement with the results obtained here. A possible explanation of these relationships could be the effects of oxidative stress on the nervous system. The brain has a high rate of oxygen consumption and is rich in lipids, which contributes to the susceptibility of its cells to oxidative stress (64). The resulting inflammatory processes (65) alter the function of serotonin and dopamine, leading to symptoms of anxiety and depression (66–68), both of which are components of the items in the EE assessment (69, 70). Consequently, the directionality of the relationship between oxidative stress and EE is not yet clear. The scope of the present study is limited to demonstrating that an association exists between 8-isoprostane and EE; future work could shed more light on this relationship.

Diet is reported to modulate oxidative stress (15). Accordingly, a research work found a reduction in urinary isoprostanes and other oxidative stress biomarkers in MS patients who consumed a Mediterranean diet (71). In the present study, analysis of the eating habits of the adolescent participants revealed a positive association between 8-isoprostane and refined cereal intake. The quality of ingested carbohydrates is known to affect metabolic risk factors (72–75), which is consistent with our findings. A cross-over study in adults reported significantly higher levels of 8-isoprostane in consumers of a refined wheat diet compared to a wheat aleurone diet (76). However, other studies did not find different levels of 8-isoprostane between the consumers of ground flaxseed or wheat bran (77), whole-grain or refined-grain products (78), and whole or refined grain foods (79).

The inverse association between the oxidative stress biomarker 8-isoprostane and fish intake found in the present study is in accordance with prior reports of an inverse association between oxidative stress and fish intake (71, 80, 81) or supplementation with fish oil (80, 82, 83) or eicosapentaenoic acid or docosahexaenoic acid (81, 84, 85). Conversely, other studies have failed to find any significant associations between oxidative stress and the consumption of fish, including oil supplements (86–88). On the other hand, review articles show an inverse association between fish consumption and prevalence of MS (89) and heart failure (90, 91), which is supported by our results.

A strength of this study is that, to the best of our knowledge, a significant association between 8-isoprostane and EE, refined cereal intake, and fish intake has not been previously demonstrated in adolescents. Additionally, the study takes a holistic approach in which anthropometric and biochemical factors, emotional management, and eating habits are analyzed. Other strong points include the multicenter design and the use of a standardized protocol, which reduces information bias. The limitations of the study include the small size and cross-sectional design of the study population. Also, sometimes the participants did not wear the accelerometer while practicing water activities or a sport requiring its removal (e.g., judo, basketball). Finally, blood samples were not obtained no to use invasive methods due to the age of the cohort, so we were unable to analyze 8-isoprostane in plasma, inflammatory or neurotransmitter biomarkers, which would have provided greater insight into oxidative stress. The results of the present study may contribute to the development of educational programs focused on the establishment of healthier lifestyles in early life stages. The findings also indicate that more research is needed to understand the interaction between food choices, emotion management, and oxidative stress status in adolescents with good or poor metabolic health.

In conclusion, a significant positive association between 8-isoprostane and EE, refined cereal intake, indicators of adiposity (BMI z-score, WHtR z-score, body fat percentage), and MS, and a negative association between 8-isoprostane and fish intake in adolescents has been found. This shows that dietary patterns such as those exhibited in the Mediterranean diet could help to prevent cardiometabolic diseases.

## Data availability statement

The datasets presented in this article are not readily available because there are restrictions on the availability of the data for the SI! Program study, due to signed consent agreements around data sharing, which only allow access to external researchers for studies following project purposes. Requests to access the datasets should be directed to Steering Committee gsantos@fundacionshe.org, rodrigo.fernandez@cnic.es, juanmiguel.fernandez@cnic.es, lamuela@ub.edu.

## Ethics statement

The studies involving humans were approved by the Ethics Committee of Instituto de Salud Carlos III in Madrid (CEI PI 35_2016), the Fundació Unió Catalana d’Hospitals (CEI 16/41), and the University of Barcelona (IRB00003099). The studies were conducted in accordance with the local legislation and institutional requirements. Written informed consent for participation in this study was provided by the participants’ legal guardians/next of kin.

## Author contributions

RL-R, GS-B, JF-A, and RF-J designed the study, project administration, and funding acquisition. SR-G, EL-S, PB, AC-G, MdM, JF-A, and AT-R supervised the study implementation and data collection. SR-G performed statistical analysis. SR-G, AT-R, and RL-R contributed to writing–original draft manuscript. SR-G, RL-R, AT-R, EL-S, JM, PB, AC-G, JF-A, RF-J, JM-G, AR-L, and RE contributed to writing–interpreted the study results, review, and editing. All authors contributed to the article and approved the submitted version.

## Funding

The SI! Program for Secondary Schools trial was supported by the SHE Foundation, the la Caixa Foundation (LCF/PR/CE16/10700001), the Fundació la Marató de TV3 (grant number 369/C/2016). Support was also provided by the Ministerio de Ciencia, Innovación y Universidades (PID2020-114022RB-I00), CIBEROBN from the Instituto de Salud Carlos III (ISCIII) from the Ministerio de Ciencia, Innovación y Universidades (AEI/FEDER, UE), and Generalitat de Catalunya. The CNIC is supported by the ISCIII, the Ministerio de Ciencia e Innovación (MCIN) and the Pro CNIC Foundation, and is a Severo Ochoa Center of Excellence (grant CEX2020-001041-S funded by MICIN/AEI/10.13039/501100011033), and INSA-UB a María de Maeztu Center of Excellence (grant CEX2021-001234-M funded by MICIN/AEI/FEDER, UE). RF-J is recipient of grant PI22/01560 funded by the ISCIII and co-funded by the European Union. GS-B was the recipient of grant LCF/PR/MS19/12220001 funded by la Caixa Foundation (ID 100010434). AT-R is a Serra Húnter fellow. EL-S was a FI-SDUR (EMC/503/2021) fellowship from the Generalitat de Catalunya. JM-G is a recipient of grant FPU21/04891 (Ayudas para la formación de profesorado universitario, FPU-2021) from the Ministerio de Educación, Cultura y Deporte.

## Conflict of interest

The authors declare that the research was conducted in the absence of any commercial or financial relationships that could be construed as a potential conflict of interest.

## Publisher’s note

All claims expressed in this article are solely those of the authors and do not necessarily represent those of their affiliated organizations, or those of the publisher, the editors and the reviewers. Any product that may be evaluated in this article, or claim that may be made by its manufacturer, is not guaranteed or endorsed by the publisher.

## Supplementary material

The Supplementary material for this article can be found online at: https://www.frontiersin.org/articles/10.3389/fnut.2023.1216445/full#supplementary-material

## Abbreviations

BMI: body mass index
BP: blood pressure
DBP: diastolic blood pressure
EE: emotional eating
HDL-c: high density lipoprotein cholesterol
LDL-c: low-density lipoprotein cholesterol
MS: metabolic syndrome
SBP: systolic blood pressure
WC: waist circumference
WHtR: waist-to-height ratio
