# Supplementary material for: Metabolic syndrome, adiposity, diet, and emotional eating are associated with oxidative stress in adolescents
Source: Front Nutr. 2023 Sep 12;10:1216445. doi: 10.3389/fnut.2023.1216445 (PMC10543258; doi:10.3389/fnut.2023.1216445)
Supplement: Supplementary file 1 [file Data_Sheet_1.pdf]

Supplementary Materials

Figure S1 – Process of the methodology used in the present study

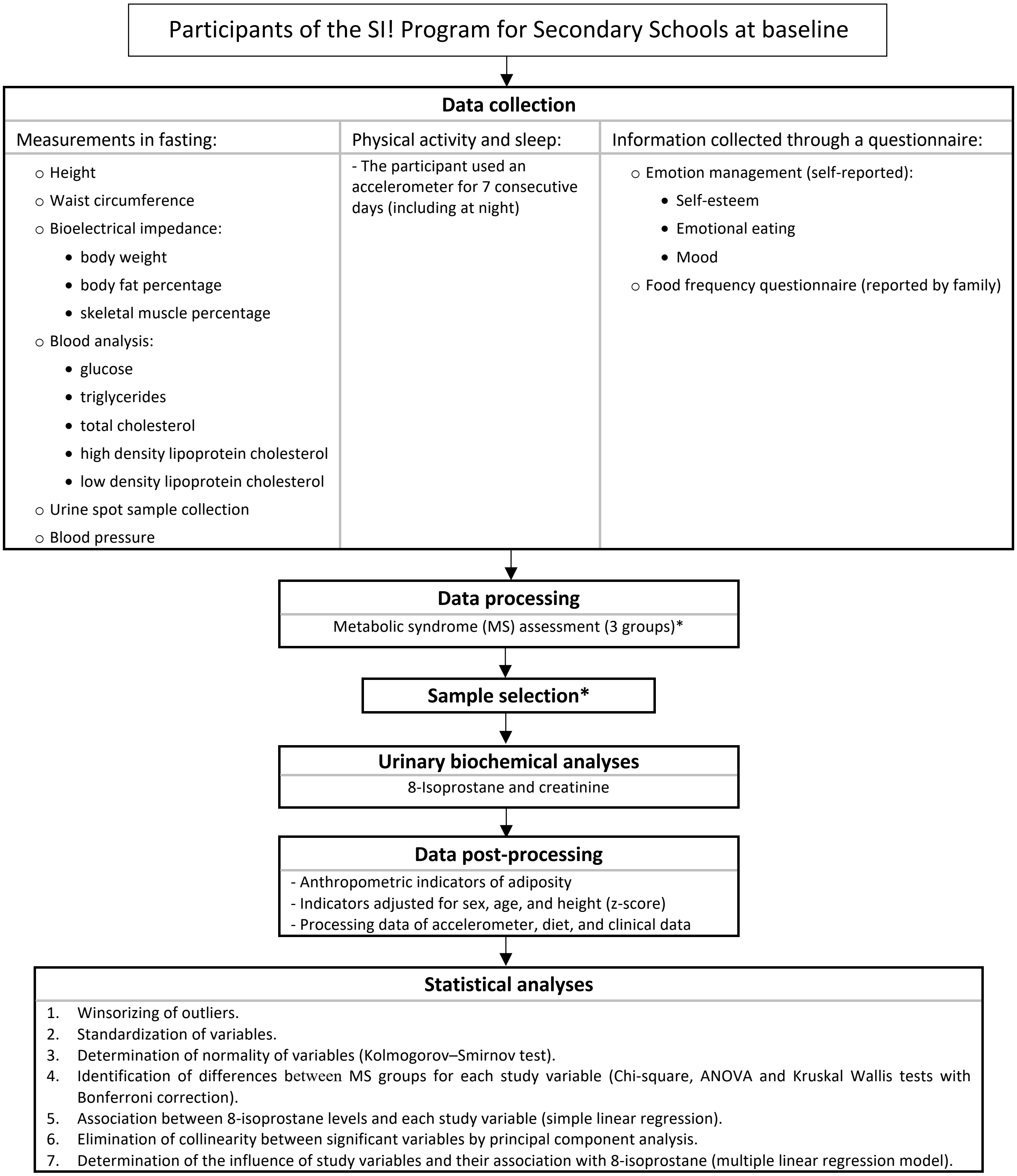

* See Figure S2.

Figure S2 – Sample selection

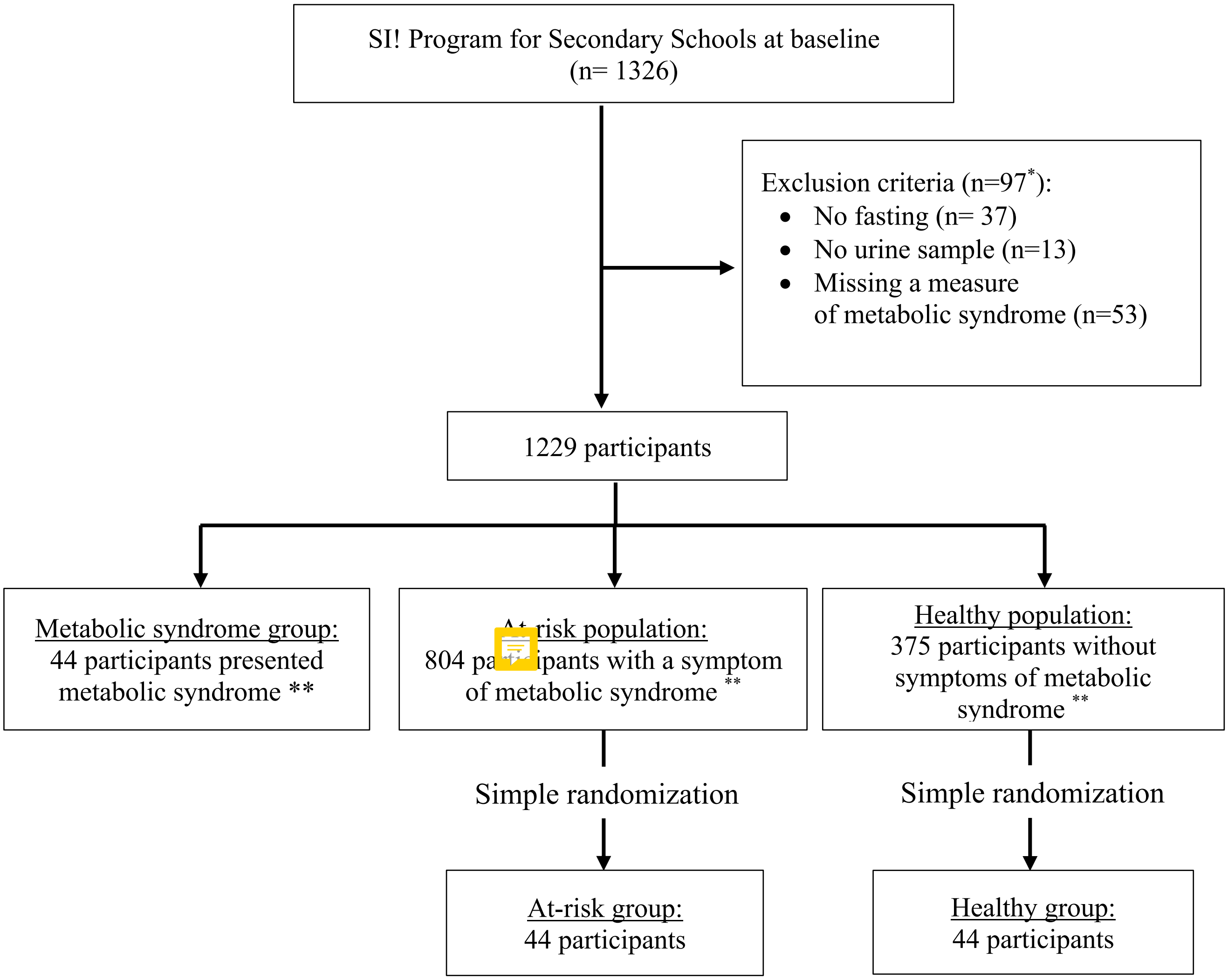

* Some participants had more than one exclusion criteria

** Further explanation is provided in the Methods section.

Table S1. – Description and comparison of the metabolic groups by sex

	Healthy		At-risk		Metabolic Syndrome	
Sex (%)	Girls (56.8)	Boys (43.2)	Girls (52.3)	Boys (47.7)	Girls (36.4)	Boys (63.6)
Age	11.8 ± 0.4	12.0 ± 0.2	12.0 ± 0.4	12.1 ± 0.5	12.2 ± 0.4	12.3 ± 0.6
Anthropometric variables:						
BMI z-score	0.29 ± 0.48	0.22 ± 0.57	1.36 ± 0.85	1.22 ± 0.90	2.26 ± 0.46	2.51 ± 0.49
WC z-score	-0.04 ± 0.47	-0.24 ± 0.68	0.91 ± 0.67	0.81 ± 0.84	1.67 ± 0.29	1.83 ± 0.26
WHtR z-score	-0.49 ± 0.54	-0.46 ± 0.65	0.45 ± 0.88	0.49 ± 0.94	1.51 ± 0.32	1.71 ± 0.35
Body fat (%)	21.7 ± 5.4†	17.7 ± 4.8†	30.1 ± 8.2†	24.3 ± 9.0†	38.2 ± 4.1	35.9 ± 4.4
Skeletal muscle (%)	34.5 ± 2.0†	36.6 ± 2.5†	31.8 ± 2.6†	35.6 ± 3.4†	28.9 ± 1.6†	31.6 ± 1.91†
Biochemistry analysis:						
Total cholesterol (mg/dL)	151 ± 23.8	142 ± 24.2	144 ± 35.7	153 ± 44.5	161 ± 48.0	166 ± 40.2
Blood glucose (mg/dL)	92 ± 6.6	89 ± 6.9	105 ± 12.9	102 ± 10.8	109 ± 7.5	113 ± 10.4
Triglycerides (mg/dL)	66 ± 10.2	66 ± 12.7	92 ± 34.7	89 ± 41.6	133 ± 71.0	166 ± 108
HDL-c (mg/dL)	66 ± 10.9	66 ± 17.9	57 ± 17.5	52 ± 17.3	56 ± 15.4	47 ± 14.0
LDL-c (mg/dL)	72 ± 24.2	64 ± 14	69 ± 27.9	83 ± 39.7	78 ± 32.9	86 ± 39.3
Urinary 8-isoprostane (pg/mg)	1003 ± 442	880 ± 379	1015 ± 442	1263 ± 425	1273 ± 602	1284 ± 517
Blood pressure:						
SBP z-score	-0.20 ± 0.96	0.18 ± 0.61	1.20 ± 0.90	1.15 ± 0.89	0.91 ± 1.01	1.40 ± 0.85
DBP z-score	0.34 ± 0.79	0.10 ± 0.82	1.05 ± 0.81	0.73 ± 1.10	1.08 ± 1.07	1.19 ± 1.01
Physical activity and sleep:						
MVPA (minutes)	79.7 ± 23.1†	63.3 ± 21.0†	73.0 ± 29.3	65.4 ± 26.1	71.9 ± 13.6	71.1 ± 21.2
Sleep duration (hours)	7.71 ± 0.66	7.99 ± 0.75	7.88 ± 0.81	7.55 ± 0.64	7.38 ± 1.05	7.03 ± 0.94
Sleep efficiency (%)	93.0 ± 2.75	92.6 ± 3.02	93.3 ± 2.86	92.3 ± 3.17	93.0 ± 3.31†	90.4 ± 2.68†
Awakenings (Frequency)	16.3 ± 5.2	17.6 ± 5.54	16.4 ± 6.98	15.5 ± 4.98	12.2 ± 5.76	15.6 ± 4.33
Awake length (minutes)	2.02 ± 0.35	2.05 ± 0.42	1.99 ± 0.36	2.14 ± 0.40	2.62 ± 0.95	2.64 ± 0.63
Emotion management:						
Satisfaction: self-esteem (1 to 4)	3.6 (2.0 - 4.0)	3.6 (1.4 – 4.0)	3.6 (3.0 - 4.0)	3.6 (2.0 - 4.0)	3.4 (0.8 - 4.0)	3.4 (1.6 – 4.0)
Emotional eating						
No emotional eating (%)	56.0	63.2	65.2	38.1	37.5	39.3
Low emotional eating (%)	16.0	21.1	13.0†	47.6†	25.0	17.9
High emotional eating (%)	28.0	15.8	21.7	14.3	37.5	42.9
Mood						
Positive mood (%)	92.0	94.7	95.7	85.7	81.2	89.3
Negative mood (%)	8.0	5.3	4.3	14.3	18.8	10.7
Dietary:						
Energy intake (kcal/d)	2284 ± 531†	2670 ± 596†	2467 ± 709	2507 ± 661	2370 ± 465	2608 ± 562
- Carbohydrates (% EI)	40.2 ± 6.5	39.6 ± 5.4	40.7 ± 6.2	42.9 ± 7.7	38.8 ± 5	41.5 ± 7.9
- Total fat (% EI)	40.6 ± 5.5	39.8 ± 4.1	40.4 ± 5.5	38.4 ± 7.4	41.7 ± 4.9	38.7 ± 7.1
- Protein (% EI)	19.2 ± 3.2	20.6 ± 3.2	18.8 ± 3.1	18.7 ± 2.8	19.5 ± 2.1	19.8 ± 3.3
Intake by groups of foods:						
Fruits and vegetables						
- Vegetables (s/d)	2.0 (0.0 - 5.3)	1.4 (0.1 - 5.8)	2.3 (0.6 - 5.9)	1.9 (0.2 - 6.1)	2.0 (0.7 - 3.7)	2.2 (0.5 - 6.5)
- Fruits (s/d)	1.3 (0.6 - 7.4)	1.6 (0.1 - 4.1)	1.7 (0.5 - 8.4)	1.9 (0.1 - 8.7)	1.2 (0.1 - 2.8)	2.1 (0.1 - 6.9)
Cereals						
- Whole grain (s/d)	0.1 (0.0 - 1.6)	0.1 (0.0 - 1.0)	0.2 (0.0 - 1.3)	0.1 (0.0 - 1.0)	0.1 (0.0 - 0.7)	0.2 (0.0 - 1.3)
- Refined cereals (s/d)	0.6 (0.0 - 1.4)	0.9 (0.1 - 1.9)	0.6 (0.0 - 1.3) †	1.1 (0.3 - 2.6) †	0.8 (0.2 - 1.7)	0.9 (0.2 - 1.6)
Fats and Oil (g/d)	24.9 (2.9 - 129)	20.0 (11.4 - 59.3)	20.9 (3.8 - 79.1)	28.4 (1.4 - 77.2)	21.1 (11.4 - 66.8)	20.9 (3.1 - 63.7)
Dairy products (s/d)	2.6 (1.0 - 4.0) †	3.8 (0.7 - 15.7) †	2.6 (0.0 - 4.6)	2.0 (0.0 - 7.0)	2.0 (0.5 - 5.5)	2.9 (0.0 - 6.9)
Legumes (s/w)	2.4 (0.0 - 4.9)	2.5 (0.9 - 12.0)	2.4 (0.0 - 28)	2.5 (0.0 - 6.9)	1.9 (0.0 - 6.0) †	4.0 (0.9 - 14.5) †
Eggs (s/w)	3.0 (1.0 - 7.0)	3.0 (0.0 - 7.0)	3.0 (0.0 - 3.0)	3.0 (0.0 - 6.0)	3.0 (0.5 - 7.0)	3.0 (0.5 - 5.5)
Fish (s/w)	2.0 (0.0 - 6.0)	4.0 (0.0 - 6.0)	3.0 (0.0 - 6.0)	2.0 (0.0 - 6.0)	1.2 (0.0 - 6.0)	1.0 (0.0 - 4.0)
Meat and processed meat (s/w)	10.9 (5.3 - 31.4)	16.3 (4.9 - 28.9)	14.4 (3.7 - 54.3)	14.5 (0.9 - 25.0)	16.9 (4.4 - 20.9)	15.4 (7.9 - 35.0)
Sugar-sweetened beverages (s/w)	0.5 (0.0 - 6.0)	0.9 (0.0 - 6.0)	0.5 (0.0 - 6.0)	1.0 (0.0 - 4.0)	0.7 (0.0 - 3.0)	1.0 (0.0 - 6.8)

Numerical variables were standardized prior to the statistical analysis. †p-values < 0.05 to refer differences between boys and girls, from chi-square (%) or t-test tests (mean ± SD or median and range)
